# Research recruitment and consent methods in a pandemic: a qualitative study of COVID-19 patients’ perspectives

**DOI:** 10.1186/s12874-023-01933-5

**Published:** 2023-05-11

**Authors:** Serena S Small, Erica Lau, Kassandra McFarlane, Patrick M Archambault, Holly Longstaff, Corinne M Hohl

**Affiliations:** 1 Department of Emergency Medicine, University of British Columbia, Vancouver, BC Canada; 2 Centre for Clinical Epidemiology & Evaluation, Vancouver Coastal Health Research Institute, 828 West 10th Ave, 7th Fl, Vancouver, BC Canada; 3 School of Medicine, Queen’s University, Kingston, ON Canada; 4 Department of Family Medicine and Emergency Medicine, Université Laval, Quebec City, QC Canada; 5 Centre de recherche du Centre intégré de santé et de services sociaux de Chaudière-Appalaches, Lévis, QC Canada; 6 Provincial Health Services Authority, Vancouver, BC Canada; 7 Faculty of Health Sciences, Simon Fraser University, Burnaby, BC Canada; 8 Emergency Department, Vancouver General Hospital, Vancouver, BC Canada

**Keywords:** Patient participation, COVID-19, Privacy, Methods

## Abstract

**Background:**

Virtual data collection methods and consent procedures adopted in response to the COVID-19 pandemic enabled continued research activities, but also introduced concerns about equity, inclusivity, representation, and privacy. Recent studies have explored these issues from institutional and researcher perspectives, but there is a need to explore patient perspectives and preferences. This study aims to explore COVID-19 patients’ perspectives about research recruitment and consent for research studies about COVID-19.

**Methods:**

We conducted an exploratory qualitative focus group and interview study among British Columbian adults who self-identified as having had COVID-19. We recruited participants through personal contacts, social media, and REACH BC, an online platform that connects researchers and patients in British Columbia. We analyzed transcripts inductively and developed thematic summaries of each coding element.

**Results:**

Of the 22 individuals recruited, 16 attended a focus group or interview. We found that autonomy and the feasibility of participation, attitudes toward research about COVID-19, and privacy concerns are key factors that influence participants’ willingness to participate in research. We also found that participants preferred remote and virtual approaches for contact, consent, and delivery of research on COVID-19.

**Conclusions:**

Individuals who had COVID-19 are motivated to participate in research studies and value autonomy in their decision to participate, but researchers must be sensitive and considerate toward patient preferences and concerns, particularly as researchers adopt virtual recruitment and data collection methods. Such awareness may increase research participation and engagement.

**Supplementary Information:**

The online version contains supplementary material available at 10.1186/s12874-023-01933-5.

## Abstract (summary)

Due to the COVID-19 pandemic, many research groups started conducting research activities virtually. In this study, we invited individuals who had COVID-19 to share their views about how researchers recruit patients and get their consent to participate in studies about COVID-19. Through interviews and focus groups, we found that British Columbians who had COVID-19 are motivated to participate in COVID-related studies, as long as researchers maintain usual precautions around data privacy and accommodate preferences for participation. Future studies may use these patient perspectives to make informed decisions that will increase and support patient recruitment, consent and retention in research studies.

**Supplementary Information:**

The online version contains supplementary material available at 10.1186/s12874-023-01933-5.

## Background

In December 2019, an outbreak of coronavirus disease (COVID-19) was reported in Wuhan, Hubei Province, China [1, 2]. As of 6 September 2022, 603,164,436 cases and 6,482,338 deaths have been reported to the World Health Organization [3]. Early in the pandemic, there was little information about diagnostic and therapeutic tools to treat and manage the virus. Governments and industry sponsors initiated urgent funding to support clinical research to sequence, diagnose, treat, and prevent COVID-19. Despite the need to accelerate clinical research about COVID-19, infection control procedures introduced challenges related to patient recruitment and data collection [4]. Many research teams halted in-person recruitment and consent activities due to infection prevention and control measures and had limited access to healthcare facilities. Researchers in British Columbia (BC) did not receive timely permission to recruit or follow-up COVID-19 patients by phone due to varying interpretations of the Freedom of Information and Protection of Privacy Act’s (FIPPA) privacy requirements and health authority concerns about COVID-19 patients being contacted multiple times for participation in different COVID-19 studies [5].

Some institutions transitioned to virtual methodologies and consent procedures in response to COVID-19 [6, 7]. BC’s health authorities and universities implemented REACH BC, a website that enables researchers to post recruitment opportunities to enable interested members of the public to find and register for participation in studies [8]. In addition to opt-in recruitment through REACH BC, the BC Centre for Disease Control (BCCDC) established a Consent to Contact Registry among those who tested positive for COVID-19, a recruitment model where patients provide generic consent for future contact about research opportunities [9, 10]. However, this opt-in, patient-driven model led to significant delays in subject recruitment and follow-up for researchers in our network, leading to loss of critical information about social, cultural and economic factors that may have been associated with COVID-19 outcomes, as well as patient-reported outcome measures, hindering research efforts in the early pandemic [11]. Virtual recruitment models may also deepen inequities in the healthcare system and prevent inclusive participation in COVID-19 research by excluding those who do not speak English, have hearing or reading impairments, have lower levels of health literacy, or have limited access to technology or internet, thus limiting recruitment of those already marginalized or with lower socioeconomic status [7]. These models tend to attract patients with high health literacy who trust the healthcare system and are among those who already participate in and benefit from scientific research [12–14].

Under-representation and lack of generalizability systematically denies minority and vulnerable populations the opportunity to benefit medically from research, and limits our understanding of disease etiology and ability to provide safe and efficacious care to the most vulnerable [15, 16]. By limiting participation in research, patients who are not provided an equal opportunity to participate may be denied the therapeutic value of research participation, including extra consultations, more frequent monitoring, and access to state-of-the-art care that may represent an advantage over standard care [17].

A number of studies have explored ethical considerations of adapting recruitment and consent approaches during the pandemic from the perspectives of research institutions [7], research ethics board members, bioethicists [18] and researchers [19]. Given the prescriptive nature of decision-making concerning contact and recruitment for research on COVID-19, seeking patient perspectives on these issues is paramount. Our primary objective is to explore COVID-19 patients’ perspectives on participation in COVID-related research studies. This study seeks to understand (1) what factors affect COVID-19 patients’ willingness to participate in COVID-related research; (2) what are patients’ preferences for recruitment and consent approaches for COVID-related research; and, (3) how should researchers communicate COVID-related research opportunities to prospective participants.

## Methods

### Study Design

We conducted an exploratory qualitative study to gather COVID-19 patients’ perspectives about research participation in the context of the global pandemic. The Behavioural Research Ethics Board at the University of British Columbia approved this study, including the verbal informed consent procedure for this study (H21-00048), and all participants provided verbal informed consent.

### Recruitment

Our study population included adults (≥ 19 years) living in BC who self-identified as having had COVID-19. We used multiple strategies to recruit eligible participants. We endeavored to continue recruitment and data collection simultaneously to achieve data saturation, but practically recruitment ceased when no additional volunteers came forward within a reasonable timeframe. We used snowball sampling through personal contacts, for which there was an established relationship with some of the researchers prior to study initiation.

We also used convenience sampling through recruitment advertisements posted online. We posted recruitment advertisements on the COVID-19 Long-Haulers Support Group Canada Facebook group and on REACH BC to target individuals who had COVID-19. The COVID-19 Long-Haulers Support Group Canada is a private Facebook group, of which the research team are not members. We leveraged personal contacts to post the recruitment advertisement to the group on our behalf in January and February 2021. We instructed interested participants to self-identify by contacting the research team directly but were otherwise unaware of any subsequent activity on that post. REACH BC is publicly funded, web-based directory that allows British Columbians to search and volunteer for research opportunities [8]. We kept recruitment advertisements active on REACH BC from February to April 2021 for prospective participants to self-identify through the website, who we then contacted via email. We included eligibility criteria on the recruitment materials and confirmed eligibility during initial contact with prospective participants. When prospective participants met eligibility criteria, we invited them to attend a focus group session, or a one-on-one interview if they were unavailable for the focus group. For participants recruited through convenience sampling, there was no prior relationship, aside from contact for recruitment purposes.

### Data collection

We used a discussion guide during the semi-structured focus groups and interviews, which the research team developed collaboratively to address themes related to research participation among COVID-19 patients (Supplementary Material 1). A trained qualitative researcher (SSS) drafted the discussion guide, which other team members revised to offer different disciplinary perspectives.

During the focus groups and interviews, we asked participants to discuss COVID-19 research and recruitment methods, and other topics related to research participation like consent and recruitment processes. We allowed participants to engage in open dialogue and ask questions beyond the discussion guide. We held focus groups and interviews virtually using the Zoom videoconferencing platform. The qualitative researcher (SSS) led the focus groups and interviews. We provided participants with study information, including background and study rationale, and consent forms prior to each session and obtained verbal consent at the beginning of each session. We audio recorded all sessions, which a research assistant (KM) transcribed verbatim.

### Analysis

We coded and thematically analyzed transcriptions using *NVivo* qualitative data analysis software (QSR International, version 12, 2022). Two analysts (EL, SSS) independently coded all transcripts inductively, and then met to discuss and revise the preliminary coding structure. Other team members (PA, CMH, HL, KM) used the codebook to analyze at least two randomly selected transcripts and provide feedback. The two analysts (EL, SSS) reconvened to discuss and revise the first iteration of the coding structure. We re-circulated the revised coding structure to the team, and with their approval, we (EL, SSS) independently re-coded all transcripts again according to the revised coding structure (Supplementary Material 2). One analyst (EL) compared both applications of the coding structure and reconciled any differences in interpretation through discussion. We then developed thematic summaries of each coding element, which the team reviewed for final comment and feedback. In the presentation of our analyses, we primarily use verbal counting to convey proportion of participants in lieu of percentages due to the small sample size (Table 1) [20].

**Table 1:** Defining verbal counting in presentation of results

Categorization	Range
A few	1 to 4 participants
Some	5 to 8 participants
Many	9 to 12 participants
Most	13 to 16 participants

## Results

### Participant characteristics

We recruited 22 individuals, of whom 16 attended a focus group or interview. Five volunteers did not meet eligibility criteria and we were unable to coordinate scheduling with one volunteer. Of those who participated, we recruited 9 from REACH BC, 4 from Facebook, and 3 from snowball sampling. The majority of participants were women (11/16). Participants recruited from REACH BC provided their date of birth as part of the registration process. The median participant age for those recruited from REACH BC was 47, ranging from 23 to 78 years old. More than half of participants reported having a background or education in research, science, or healthcare, and/or having previously participated in research. Many are early pandemic survivors.

From February to April 2021, we held two focus groups with six and four participants, respectively, followed by six one-on-one interviews. Focus groups and interviews were 20 to 67 min in duration. Participants described variable experiences with COVID-19, in terms of severity, duration, timing, and use of medical services. Many participants reported having contracted COVID-19 in the first year of the pandemic when limited diagnostics and treatment were available. Most self-managed at home or sought alternative avenues of care (e.g., telehealth or family doctor) and only one participant reported having gone to the emergency department. A few participants described long-term effects they attributed to COVID-19, including fatigue and an inability to return to routine activities.

### Factors affecting Research Participation among COVID-19 patients

Participants identified key factors that would affect their willingness to participate in different types of research about COVID-19, including approaches to recruitment and consent. We conceptualize these factors thematically in terms of autonomy and the feasibility of participation, overarching attitudes toward research about COVID-19, and privacy concerns and protections, summarized in Table 2 and addressed in greater detail below.

**Table 2:** Summary of key findings

Theme	Sub-themes
Autonomy and feasibility of participation	- Decision of when and how to participate - Control data - Time and frequency - Recovery stage and health status
Attitude toward research on COVID-19	- Interest in research - Altruism
Privacy concerns and protections	- Misuse - De-identification

#### Autonomy and feasibility of participation

Autonomy in participation was an emerging theme from discussions about participation in COVID-related research. Many participants discussed elements of autonomy in participation, in terms of wanting to be able to opt-in and opt-out of research themselves, removing data from a study, deciding the frequency, timing, and format of participation, and determining who could use their data. This theme was present across discussions of all types of research and was particularly the case in the context of research on hospital records and having multiple researchers contact them to participate in various studies.

The feasibility of participation also affected participants’ willingness to engage in COVID-related research. Time was a primary concern for many participants, especially for interview-based research, whether time of day or week and duration or time required. When discussing the prospect of participating in multiple studies, participants also considered the frequency of participation. Similarly, the location of the research was likely to have an impact on the participants’ willingness and ability to participate. Ultimately, participants wanted to evaluate the practicalities of research participation and whether participating fit their schedules and preferences. Respect for time and location were related, with a few participants highlighting the need to manage scheduling if travel was required.

*“I find like, there should be some appreciation for – for the patients and the public who give their time for the research…it’s really hard to make the time in a work day, so you actually have to take time out of your work day.”* (Participant 6)

*“…as long as our time is respected, my time is respected. As long as I know what I’m getting into at the start of it, then I don’t feel like it’s terribly burdensome. I agree with Participant 9, I don’t want to receive a call at 7 o’clock in the morning or at 10 o’clock at night.”* (Participant 7)

While autonomy and feasibility of participation were associated with participation in other research, they are particularly vital for COVID-19 patients who may be at various recovery stages and health statuses at the time of the research. A few participants discussed the impact of long-term COVID-19 symptoms on their physical ability to complete tasks, while a few others were concerned with the psychological aspect of recovery. The burdens of time and recovery stage co-occurred, with a few participants noting that sensitivity to health status was necessary in considering time to participate.

*“…ask people what’s the best time of day to talk to you, because some of us are better in the morning, some rest all day and we have a good hour at 3:00, others it’s in the evening.”* (Participant 3)

*“…I think it does matter where somebody is in their recovery, and we’re all in different places…it’s really unpredictable for a lot of us. Like when we wake up in the morning – we don’t know what kind of day we’re going to have, we don’t know necessarily what we’re going to be able to handle on that day until we’re actually going through it. It’s very hard to plan anything because of that.”* (Participant 4)

#### Attitude toward Research on COVID-19

Participants with a more positive perception toward or interest in research were more likely to state that they would be willing to participate in various types of COVID-19-related research. This was driven, at least in part, by the perceived impact of participating, which participants viewed as producing both personal and social benefits.

On a personal level, participants discussed having an interest in research and that participating in research offered opportunities to feel represented and to find a positive outcome from a negative experience. For some participants, a positive attitude toward research also translated to a lower level of concern about being contacted by multiple researchers.

*“…just to know that people are using my data could be very validating for some of us…it would help with that feeling of being counted in some way.”* (Participant 4)

*“I think that COVID was like, for me personally, a really negative experience. Especially being in isolation and – and when you come out of it…research is a great way to kind of make some positive out of the experience...”* (Participant 8)

On a social level, most participants discussed research participation as an altruistic act and a civil duty. Many participants also viewed COVID-19 as an extraordinary time, which increased their willingness to participate in research about COVID-19 compared to other topics. This was particularly the case for research on hospital records. Participants emphasized the importance of research on COVID-19 to address the seriousness of the pandemic and to capture differences among the various population sub-groups who had COVID-19.

*“Yeah, I’m pretty busy so [being contacted multiple times] may – it may wear on you a little bit…But, I mean, you know with something as new as this, you also want to help too. I think it’s your civic duty…to help out and try to get through this.”* (Participant 12)

*“I think it’s important to be a part of that kind of research, because it has the potential to help others.”* (Participant 13)

#### Privacy concerns and protections

Some participants were concerned with potential misuse of personal health information collected for research on hospital records or biological samples. Participants discussed misuse in terms of having their health information used against them to affect employment, insurance, or ability to travel, sold or commercialized, or used for illegitimate research purposes.

*“I would want to know how the information is going to be used, and where it’s going to be stored.”* (Participant 1)

*“I would like to evaluate who’s doing the research, how they’re doing it, and what they’re doing with that information before I’m giving access to it, because there’s all types of people that research.”* (Participant 9)

Some participants said that de-identification, as is current practice, was necessary if researchers were using their personal health information without consent, but many also felt that de-identification would increase their comfort in participating in research even if they had provided consent.

*“I think my main hesitation with [research on my records] would be if it was tied to more personal information about me, or more of my medical history. If it’s just statistical information…if it doesn’t tie it to me – as much, then that’s a little…less unnerving.”* (Participant 13)

*“As long as it’s anonymous I don’t care, ‘cause I know how much work it is to get the data...”* (Participant 3)

### Preferences for Consent and Mode of Contact

We asked participants how they felt about privacy rules in BC that some institutions interpreted as prohibiting researchers from contacting hospitalized patients directly for research purposes without their prior consent and by other institutions as permitting Consent to Contact. A few participants had favorable views toward Consent to Contact. Although only one participant expressly stated that they disagreed with it, a few others articulated views consistent with this position, for example, by stating that the researchers could call them and obtain consent over the phone. One participant preferred a centralized approach like the BCCDC’s Consent to Contact Registry.

*“…I think that what the BCCDC is doing now, reach out to people who’ve tested positive, is a good way… they were just calling people to say ‘do you consent to be contacted?’.”* (Participant 7)

We asked some participants whether the circumstances of the pandemic would change their acceptance of broader consent and their perspectives on obtaining Consent to Contact. Participants had split views toward this topic, with four out of seven indicating that they would give exemptions given the unique circumstances of the pandemic. A few participants in opposition to changing the rules highlighted alternative modes of obtaining consent for future contact.

*“I completely understand informed consent, consent to contact and the importance [of] it, but I do think that in times like COVID, you have to re-assess what the framework is – because that’s what we’ve been doing the whole time, is re-assessing what we have, what works and what doesn’t, and what we can change during these times.”* (Participant 8)

### Facilitating recruitment & increasing Research Participation among COVID-19 patients

Participants discussed ways to communicate research opportunities and strategies to reduce the burden of participation among COVID-19 patients, focusing on maintaining the feasibility and autonomy of participation.

Participants suggested approaches to communicate research opportunities, which aligned with the ways that they heard about the current study. Some participants mentioned REACH BC, and a few mentioned other academic networks, posters in healthcare settings, and community-based nurses. Regarding the preferred mode of communication, some participants favored emails, citing the flexibility of being able to respond on their own time, and some mentioned social media, news media, or letters.

*“Well, I – I guess it depends on the age group. Because if you’re thinking about young people, obviously the best way to reach would be to advertise on social media…”* (Participant 11)

*“…REACH BC is a really good platform where people can sign up and volunteer…”* (Participant 14).

To reduce the burden of participation, many participants wanted autonomy and the ability to choose scheduling and response modality, as well as the choice to participate in general by being able to opt out or withdraw from studies. A few participants wanted to receive advanced notice of the expectations of their participation, study procedures, and questions.

*“…it’s helpful to know exactly what to expect, before starting.”* (Participant 1)

*“…I would assess how beforehand if there was going to be a burden or not, and then make a decision. Like, is this going to stress me out, how tired am I? And…the option to say ‘I’m actually not going to do it’ ...”* (Participant 9)

The characteristics of those conducting the research were also a consideration. Participants highlighted the need for researchers to be sensitive to trauma, to build trust, to use plain language, and to show appreciation to participants.

*“I think sensitivity around the fact that this is – has been a very traumatizing illness for a lot of us, would be the key thing for me.”* (Participant 4).

*“So, yeah, I think there needs to be clear, plain language…”* (Participant 3).

## Discussion

To our knowledge, this is the first study to explore patients’ perspectives about consent and recruitment for COVID-19 research. Patient involvement ensured our research questions and findings were relevant and important to patients. In this study, 16 individuals who had COVID-19 provided valuable insights to inform recruitment and consent methods for future research. We identified four main findings: (1) participants want autonomy; (2) altruism and self-interest are motivating factors for participating in COVID-19 research; (3) decisions concerning consent waivers for COVID-19 research depend on privacy protection measures; and, (4) remote and virtual approaches are preferred modes of contact and research delivery for COVID-19 research.

Autonomy is the most frequently mentioned theme in this study. Participants wanted the choice to hear about research opportunities, to decide whether and how to participate in research, and to determine how their data are used. Similarly, previous studies [7, 18, 19] that examined researchers’ and research institutions’ perspectives on adapting the recruitment and consent process for COVID-19 research also support that adaptations must center on protecting the rights and autonomy of research participants. In BC, health authority interpretations of provincial privacy rules and concerns about the burden of Consent to Contact for patients who may be contacted by multiple research teams contradicted patients’ stated preferences for autonomy, as patients in the early pandemic were not given the choice to hear about and decide for themselves about whether to get involved in COVID-19 research. Our findings provide initial empirical evidence in support of reassessing interpretations of these rules and regulations, and support the need to ensure that all patients have equitable access to information about research opportunities. In addition, our research suggests that patients be given the opportunity to voice preferences about how and when they would welcome future contact.

Most participants highlighted altruism and self-interest as primary motivators for enrolling in COVID-19 research. This was consistent with results from a multi-national study reporting that altruism and personal interest were primary motives for participation in COVID-19 clinical trials. Similar results were also reported in health services research [21–23] and some researchers advocate using altruism to increase participant recruitment for biomedical research [17]. Others have argued that using altruism may be coercive and exploitative by privileging society’s interests above the needs of individual patients [23]. Our participants did not articulate this concern. The research community needs a deeper understanding of how self-interest and altruism influences participants’ willingness to participate in COVID-19 research to ensure that participants understand whether research studies will meet their expectations when giving informed consent [24]. This will also help researchers develop ethical and effective recruitment strategies for COVID-19 research.

Some participants expressed privacy concerns for waiving consent, particularly for COVID-19 research using hospital records and biological samples. However, most participants indicated they agreed with consent waivers for de-identified data. Our findings suggest that many participants lacked awareness of existing governance structures that already protect individuals’ data, especially when consent is not in place. Although there are resources to assist health organizations in developing data and information governance structures, such as the Canadian Institute for Health Information’s Health Data and Information Governance and Capability Framework [25], we are unaware of materials specifically targeting patients or potential research participants to inform them about existing protections. This gap suggests an opportunity and need to inform the public about information governance in research contexts.

Participants identified advantages and barriers to several modes of contact and research delivery. They did not seem categorically opposed to any of them but leaned towards remote and virtual approaches (e.g., telephone, email, or videoconferencing). Most participants liked having the REACH BC platform as a central hub for research opportunities, and preferred email for communications and virtual meetings for interviews. Remote, virtual approaches are advantageous to reducing the burdens of time and cost of participating. These approaches are essential in the context of the pandemic or future infectious disease outbreaks, in which participants may undergo different recovery stages or social isolation. Of note, our participants did not discuss potential challenges and consequences of remote and virtual approaches that rely on self-identification among interested volunteers with high health and technology literacy, including under-representation of specific populations, reduced data richness, limited generalizability of results, or exclusion of populations lacking access to internet and electronic devices [26]. Based on our work, researchers should pursue various methods of contact and research delivery, but further research is required to understand ways to reach under-represented and marginalized communities in the context of increasing remote and virtual research methods.

Selection bias is a major limitation of this study. Convenience sampling limited representativeness of our study sample. Our sample is geographically limited, having only recruited adults living in BC, and is not representative of visible minorities. Our sample also consists mainly of individuals with higher education and experience in research, which may lead to more favorable views toward research in general and increase their likelihood of responding positively toward being contacted for research opportunities. Similarly, by virtue of digital recruitment and data collection methods, participants likely had higher levels of digital literacy and access to a computer with Internet. Given the timing of the study, many participants are early pandemic survivors, many of whom had COVID-19 before vaccines were widely available. This may have increased the perceived urgency of research at the time compared to those who may have had COVID-19 in later waves. As a result, these findings may not be representative of the entire population affected by COVID-19 and may not be generalizable to individuals commonly inadvertently excluded from health research. Future studies should seek to assess the themes identified in this study among a broader sample in different jurisdictions through quantitative methods or using similar methods when researchers urgently seek to address a new health problem, and a limited pool of patients are available for research participation.

## Conclusions

In this study, we sought to explore COVID-19 patients’ views on research participation. We identified several considerations related to autonomy, privacy, and modes of contact. Despite patients being motivated to participate, researchers must be considerate of their preferences and concerns, while being mindful of pragmatic aspects of participation. Patient perspectives are increasingly relevant in the design and implementation of research studies, particularly as researchers seek novel ways to conduct research in the pandemic. Research recruitment methods should prioritize patient autonomy, while seeking to increase the engagement of under-represented and marginalized groups as we increasingly adapt virtual and remote methods of recruitment and data collection.

## Electronic supplementary material

Below is the link to the electronic supplementary material.

Supplementary Material 1

Supplementary Material 2

Supplementary Material 3

Supplementary Material 4

Supplementary Material 5

## Data Availability

The audio recordings and transcripts analyzed during the current study are not publicly available per the ethics approval for this study, but are available from the corresponding author on reasonable request.

## Abbreviations

BC: British Columbia
BCCDC: British Columbia Centre for Disease Control
COVID-19: Coronavirus disease
FIPPA: Freedom of Information and Protection of Privacy Act
