# Supplementary material for: Research recruitment and consent methods in a pandemic: a qualitative study of COVID-19 patients’ perspectives
Source: BMC Med Res Methodol. 2023 May 11;23:113. doi: 10.1186/s12874-023-01933-5 (PMC10173898; doi:10.1186/s12874-023-01933-5)
Supplement: Supplementary file 1 — Supplementary Material 1 [file 12874_2023_1933_MOESM1_ESM.docx]

**Canadian COVID-19 Emergency Department Rapid Response Network (CCEDRRN) Patient Focus Groups
Discussion Guide**

**Welcome and Introductions**

**[Verbal consent script]**

**Guidelines**

- No right or wrong answers; just different points of view
- Session will be recorded; reminder of confidentiality
- Listen respectfully; only one person speaks at a time

**Introduce topic:** Today we will be talking about participating in COVID-19 research. We are going to start the discussion by talking about your opinions toward different kinds of research being done about COVID-19. We will then discuss how researchers should be asking for permission to do research and some ways to support these activities.

1. If you tested positive for COVID-19, how would you feel about researchers accessing your medical charts and other hospital records without your express permission?
   1. *PROMPT:* Even if researchers don’t ask your permission, the research ethics board approves the study and data is anonymized, so your identity is protected.
2. Would you be willing to participate in research that involved taking a blood sample or other biologic samples, like urine or tissue? Why / why not?
3. How would you feel about researchers contacting you once for a short 5-15 minute interview 30 days after you left the hospital to understand your health and well-being after leaving the hospital? Why / why not? Would your opinion change depending on whether you were tested or not tested for COVID-19 in the hospital?
4. How would you feel about researchers contacting you 4 to 5 times over the course of a year after you left the hospital for a 5-15 minute interview?
5. For this type of follow up research, would you prefer to be contacted by telephone, email, text message, or letter mail?
6. Different types of studies may take longer or ask you different kinds of information. For example, a survey may ask several simple, short answer or multiple-choice questions, while an interview would ask more in-depth and open-ended questions. Would your contact preferences change depending on the type of study?
7. Would you want to be allowed to remove your personal health data from a research project?
   1. *PROMPT:* What if this affected the quality of the data and evidence generated through the research?
8. If you have children, would you feel the same way about being contacted for your children to participate in a research project?
9. Because COVID-19 is a new disease, researchers are interested in getting all kinds of different data from people with a confirmed or suspected case of COVID-19 to answer questions about how you experienced the disease, what treatments you had and so on. Do you have any concerns about multiple different researchers using your data to conduct research about COVID-19?
   1. Do you have any concerns about receiving multiple phone calls from multiple research groups about different studies?
   2. Do you have any concerns about one researcher calling to present multiple studies that you could participate in?
10. Usually researchers work on site in hospitals to ask patients permission to contact them for research purposes because healthcare providers are often too busy to ask. Because of privacy laws in BC, researchers and other people outside of your circle of care aren’t allowed to contact you for research purposes without first getting your consent – meaning you have to agree to be contacted by the research team after you leave the hospital. To what extent do you agree or disagree with this rule?
    1. Because of the COVID-19 pandemic, researchers aren’t allowed to be on site to ask for patient consent, but healthcare providers are too busy to do these kinds of activities. This law means that some researchers may not be able to contact patients that had COVID-19 to answer research questions. Does this situation change your opinion about getting consent to contact patients?
    2. How do you think that researchers can balance privacy concerns with the need to collect important data about people affected by COVID-19?
11. How would you like to hear about opportunities to participate or volunteer as a study subject in COVID-19 research? Thinking about the different types of research we talked about earlier, would your opinion on this change depending on the type of research study being done?
12. [*IF FOCUS GROUP*: By show of hands,] Have you heard of the REACH BC platform?
    1. [*IF YES*] What do you know about it?
    2. [*ELABORATE IF REQUIRED*] REACH BC was developed as part of Canada’s Strategy for Patient-Oriented Research, or SPOR, which aims to shift patients, caregivers, and families from passive recipients of health services into proactive partners who help shape health research and healthcare. The REACH BC platform allows patients to search for and browse current health research opportunities, allows researchers to post calls for participation, and connect the two through a secure online platform.
    3. What do you think are some of the advantages to having a platform like this?
    4. What about some disadvantages?
       1. *PROMPT*: Do you think everyone in BC or Canada affected by COVID-19 would feel the same as you? Why / why not?
13. Open floor to any other questions or discussion points.
