# Supplementary material for: Research recruitment and consent methods in a pandemic: a qualitative study of COVID-19 patients’ perspectives
Source: BMC Med Res Methodol. 2023 May 11;23:113. doi: 10.1186/s12874-023-01933-5 (PMC10173898; doi:10.1186/s12874-023-01933-5)
Supplement: Supplementary file 2 — Supplementary Material 2 [file 12874_2023_1933_MOESM2_ESM.docx]

**Research recruitment and consent methods in a pandemic: A qualitative study of COVID-19 patients’ perspectives
Supplementary Materials: Final Coding Schema**

| **Name of Code** | **Node** | **Sub-Node** | **Description** |
| --- | --- | --- | --- |
| Participant characteristics |  |  | Participants provide information about self |
|  | COVID experience |  | Participants’ experience with COVID-19, including care sought and long-term impact |
|  | Research experience |  | Participants’ previous experience in research, COVID-related or otherwise |
|  | Drivers for research participation |  | Participants’ rationale for participating in research, including this research |
|  | Professional or educational background |  | Participants’ professional or educational backgrounded produced during discussion |
| Barriers and facilitators to research participation |  |  | Participants describe factors that would support (facilitators) or hinder (barriers) their willingness to participate in research |
|  | Barriers |  | Factors that would have a negative effect on willingness to participate |
|  |  | Burden of research | Factors that were viewed as burdensome in the context of participation (e.g., location, recovery stage, time) |
|  |  | Legitimacy of researchers | Perceptions of the legitimacy of the researchers undertaking the research |
|  |  | Misuse of personal health information | Concerns about the handling and use of personal health information |
|  |  | Stigma | Concerns about potential judgment or discrimination |
|  |  | Other privacy concerns | Other privacy concerns (aside from misuse or stigma) |
|  | Facilitators |  | Factors that would have a positive effect on willingness to participate |
|  |  | Attitude toward research | Perceptions about research in general |
|  |  | Autonomy | Perceptions about participants’ ability to make own decisions regarding participation |
|  |  | De-identification | Perceptions about de-identification of health information in research |
|  |  | Impact of participating | Perceptions about impact of participation, including public good / altruism |
|  |  | Patient engagement | Perceptions about patient’s (participants’) ability to inform research |
|  |  | View COVID as an extraordinary time | Perceptions that pandemic is an extraordinary time |
| Consent |  |  | Perceptions of consent in different contexts |
|  | Consent to contact |  | Requirements for research to obtain patient’s consent in hospital to be contacted by researchers in future |
|  | Consent to use data |  | Need for and provision of consent for researchers to use participants’ data, including accessing medical charts or records |
|  | Consent for multiple research projects |  | Need for and provision of consent in context of multiple researchers using data (e.g., broad consent, consent at each time) |
| Hearing about research opportunities |  |  | Participants discuss different ways of hearing about research opportunities |
|  | Current modes of contact |  | Ways in which participants are currently contacted (or not) for research opportunities |
|  | Email |  | Researchers contact patients about research study via email |
|  | Hospital discharge follow up |  | Researchers contact patients about research study after discharge from hospital |
|  | REACH BC |  | Research advertisements on REACH BC |
|  | Social media |  | Research advertisements on social media (e.g., Facebook, Instagram) |
|  | Other |  | Research advertisements through other channels (including radio, text message, phone) |
|  | Concerns |  | Concerns of being contacted in any modality (including absence of concerns) |
| Study type |  |  | Type of research participants refer to in discussions |
|  | Biological samples |  | Research involves collection of biological samples (e.g., blood, urine, saliva) |
|  | Follow up interviews |  | Research involves interviews following hospital discharge |
|  | Hospital records |  | Research involves using information from patient’s hospital records |
|  | Other |  | Other research type, or research in general |
| Strategies to reduce research burden |  |  | Perspectives of potential strategies to reduce burden of research participation |
|  | Accessible language |  | Provide information in accessible and easy to understand language |
|  | Advanced notice |  | Give participants study information and notice in advance |
|  | Patient engagement |  | Enable patients to help inform research |
|  | Provide questions in advance |  | Give participants the questions that will be asked in advance |
|  | Provision of choice |  | Include options for scheduling, response format, decline / opt-out, being in control |
|  | Researcher compassion, sensitivity, or appreciation |  | Characteristics of those conduct the research |
