# Supplementary material for: Research recruitment and consent methods in a pandemic: a qualitative study of COVID-19 patients’ perspectives
Source: BMC Med Res Methodol. 2023 May 11;23:113. doi: 10.1186/s12874-023-01933-5 (PMC10173898; doi:10.1186/s12874-023-01933-5)
Supplement: Supplementary file 3 — Supplementary Material 3 [file 12874_2023_1933_MOESM3_ESM.docx]

GRIPP2 Short Form

| **Section and topic** | **Item** | **Reported on page no.** |
| --- | --- | --- |
| 1: Aim | Report the aim of PPI in the study | 3 |
| 2: Methods | Provide a clear description of the methods used for PPI in the study | 3-5 |
| 3: Study results | Outcomes – Report the results of PPI in the study, including both positive and negative outcomes | 5-10 |
| 4: Discussion and conclusions | Outcomes – Comment on the extent to which PPI influenced the study overall. Describe positive and negative effects | 11 |
| 5: Reflections / critical perspective | Comment critically on the study, reflecting on the things that went well and those that did not, so others can learn from the experience | 12 |
